# The Delay in the Development of Experimental Colitis from Isomaltosyloligosaccharides in Rats Is Dependent on the Degree of Polymerization

**DOI:** 10.1371/journal.pone.0050658

**Published:** 2012-11-29

**Authors:** Hitoshi Iwaya, Jae-Sung Lee, Shinya Yamagishi, Aki Shinoki, Weeranuch Lang, Charin Thawornkuno, Hee-Kwon Kang, Yuya Kumagai, Shiho Suzuki, Shinichi Kitamura, Hiroshi Hara, Masayuki Okuyama, Haruhide Mori, Atsuo Kimura, Satoshi Ishizuka

**Affiliations:** 1 Graduate School of Agriculture, Hokkaido University, Sapporo, Japan; 2 Research Faculty of Agriculture, Hokkaido University, Sapporo, Japan; 3 Graduate School of Life and Environmental Sciences, Osaka Prefecture University, Osaka, Japan; National Institute of Agronomic Research, France

## Abstract

**Background:**

Isomaltosyloligosaccharides (IMO) and dextran (Dex) are hardly digestible in the small intestine and thus influence the luminal environment and affect the maintenance of health. There is wide variation in the degree of polymerization (DP) in Dex and IMO (short-sized IMO, S-IMO; long-sized IMO, L-IMO), and the physiological influence of these compounds may be dependent on their DP.

**Methodology/Principal Findings:**

Five-week-old male Wistar rats were given a semi-purified diet with or without 30 g/kg diet of the S-IMO (DP = 3.3), L-IMO (DP = 8.4), or Dex (DP = 1230) for two weeks. Dextran sulfate sodium (DSS) was administered to the rats for one week to induce experimental colitis. We evaluated the clinical symptoms during the DSS treatment period by scoring the body weight loss, stool consistency, and rectal bleeding. The development of colitis induced by DSS was delayed in the rats fed S-IMO and Dex diets. The DSS treatment promoted an accumulation of neutrophils in the colonic mucosa in the rats fed the control, S-IMO, and L-IMO diets, as assessed by a measurement of myeloperoxidase (MPO) activity. In contrast, no increase in MPO activity was observed in the Dex-diet-fed rats even with DSS treatment. Immune cell populations in peripheral blood were also modified by the DP of ingested saccharides. Dietary S-IMO increased the concentration of *n*-butyric acid in the cecal contents and the levels of glucagon-like peptide-2 in the colonic mucosa.

**Conclusion/Significance:**

Our study provided evidence that the physiological effects of α-glucosaccharides on colitis depend on their DP, linkage type, and digestibility.

## Introduction

Isomaltosyloligosaccharides (IMOs) are hardly digestible in the upper gastrointestinal tract, and the ingestion of isomalto-oligosaccharides containing isomaltose (9.0%), panose (21.8%), isomaltotriose (2.0%), and isomaltotetraose (7.4%) significantly increases the number of fecal bifidobacteria in humans [1]; thus, these saccharides are considered to be prebiotics. The physiological properties of indigestible saccharides likely depend on the degree of polymerization (DP). Although modifications of intestinal microflora by isomalto-oligosaccharides, Inulin-type fructans with various DPs have been investigated [2]–[4], the influence of different DPs on intestinal homeostasis has not been determined.

Inflammatory bowel disease (IBD), Crohn's disease and ulcerative colitis, is believed to result from a complex interplay of genetic and environmental factors leading to inflammation of the gut mucosa [5]. Dextran sulfate sodium (DSS)-induced colitis exhibits many pathophysiological features that are similar to IBD, including mucosal damage, ulceration, leukocyte infiltration, and inflammatory cytokine production [6], [7]. The migration of polymorphonuclear neutrophils (PMN) into the mucosa is a pathological hallmark of IBD that is commonly observed in the lamina propria [8]. Some oligosaccharide prebiotic preparations have been shown to inhibit experimental colitis and to increase luminal concentrations of organic acids, such as lactic acid [9], [10]. However, other reports show no benefit of oligosaccharides in experimental colitis [11], [12]. Thus, there are discrepancies in the potential anti-inflammatory effects of dietary non-digestible saccharides. The differences in DP of these saccharides may provide a plausible explanation for these controversial results.

Glucagon-like peptide (GLP)-1 and GLP-2 are secreted from L-cells, an enteroendocrine cell that is prevalent in the distal ileum and colon [13]–[15]. GLP secretion from L-cells is stimulated by nutrients such as sugars, amino acids, and long-chain fatty acids [16]. It is difficult for these nutrients to reach large intestine [17]. On the other hand, short-chain fatty acids (SCFAs) are produced from macrofibrous material that reaches the distal gut by bacterial fermentation. A recent report demonstrated that SCFAs produced by bacterial fermentation directly enhance the release of peptides via G-protein-coupled receptor (GPR) 43 in the gut epithelia [18]. GLP-2 administered to normal mice and rats promotes the growth of the mucosal epithelium in the small and large intestine [19], [20]. Also, GLP-2 has therapeutic potential against DSS-induced colitis in mice [21]. Hence, the increase in GLP-2 secretion potentially contributes to the prevention and/or the amelioration of IBD. Whether the physiological effects of IMOs are dependent on DP is still unknown.

**Table 1:** Diet composition.

Ingredient	Quantity (g/kg)			
	**Control**	**S-IMO**	**L-IMO**	**Dex**
Casein^1^	200.0	200.0	200.0	200.0
Soybean oil^2^	70.0	70.0	70.0	70.0
Crystallized cellulose^3^	50.0	50.0	50.0	50.0
Mineral mixture^4^	35.0	35.0	35.0	35.0
Vitamin mixture^5^	10.0	10.0	10.0	10.0
L-cystine	3.0	3.0	3.0	3.0
Choline bitartrate	2.5	2.5	2.5	2.5
S-IMO		30.0		
L-IMO			30.0	
Dextran 200,000 (Dex)				30.0
Sucrose^6^	To make 1 kg			

1 NZMP Acid Casein (Fonterra. Ltd., Auckland, New Zealand).

2 (J-Oil Mills Tokyo, Japan).

3 Ceolus PH102 (Asahi Chemical Industry, Tokyo, Japan).

4,5 Mineral and vitamin mixtures were prepared according to the AIN93-G formulation.

6 Nippon Beet Sugar Manufacturing Co., Ltd., Japan).

We prepared two types of IMOs with different DPs: short-sized IMO (S-IMO; average DP = 3.3) and long-sized IMO (L-IMO; average DP = 8.4). Dextran (Dex) has a large DP compared with S-IMO and L-IMO. Here, we examined the differences in the dietary effects of different IMOs and Dex on DSS-induced colitis using a rat model.

**Table 2:** Disease activity index^1^.

Score	Weightloss (%)	Stool consistency	Rectal bleeding
0	None	Normal	None
1	1∼5		
2	5∼10	Pasty stool	Occult bleeding
3	10∼20		
4	>20	Diarrhea	Gross bleeding

1 DAI is a cumulative score calculated from weight loss, stool consistency, and occult bleeding as shown above.

## Materials and Methods

### Rats and Diets

This study was approved by the Hokkaido University Animal Committee (Permit Number: 08–0130), and the animals were maintained in accordance with the Hokkaido University guidelines for the care and use of laboratory animals. Four-week-old male Wistar rats (Japan Slc, Inc., Shizuoka, Japan) were housed individually in stainless steel cages with wire-mesh bottoms. The cages were located in a room with controlled temperature (22±2°C), relative humidity (40–60%), and lighting (lights on 08∶00–20∶00 h) throughout the study. The test diets for the rats were based on modified versions of the American Institute of Nutrition (AIN)-93G rodent diets [22] containing either 3% S-IMO (average DP = 3.3, Table 1, Figure S1, and S2), 3% L-IMO (average DP = 8.4, Table 1, Figure S1 and, S2), or 3% Dex (Wako Pure Chemical Industries, Osaka, Japan; average DP = 1230, Table 1). The doses of IMOs and Dex in the diet were selected within the range of dietary fiber content in AIN-93 formula [22]. The rats had free access to food and deionized water.

**Figure 1:**
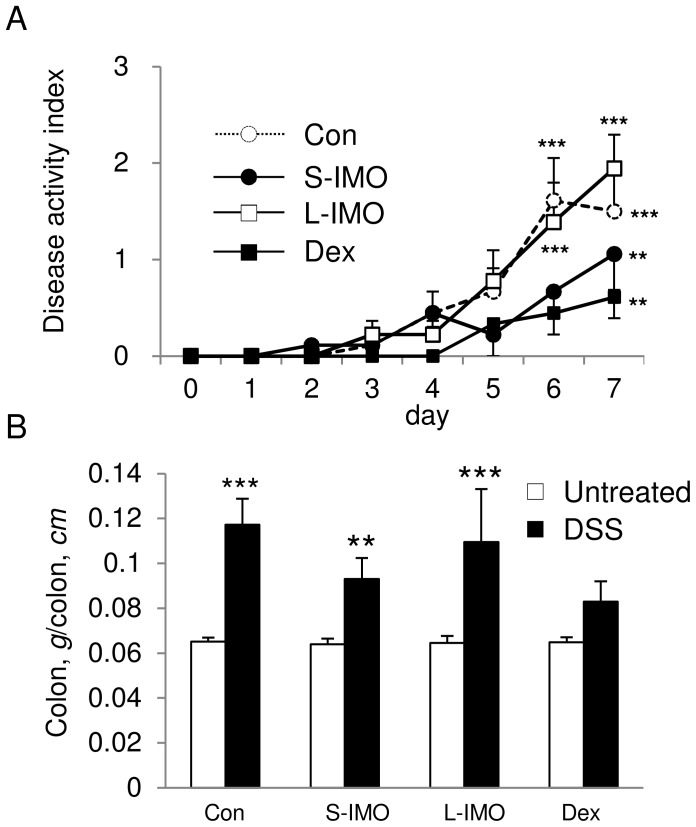
Changes in DAI score and colon weight per length. (A) DAI was calculated by the sum of three clinical scores (stool consistency, rectal bleeding, and weight loss) during the DSS treatment period. Significant differences comparing scores to values on day 0 (before DSS treatment) were determined by Dunnett’s multiple comparison test (**P<0.01, ***P<0.001). (B) Colon weight per length with or without DSS treatment. Two-way ANOVA *P* values for the colon weight per length (diet and treatment) were 0.0047 for diet, <0.0001 for treatment, and 0.0053 for the interaction between diet and treatment. No significant difference was observed for strain. Significant differences between the untreated group and the 2% DSS-treated group were determined by an unpaired two-tailed Student’s *t*-test (**P<0.01, ***P<0.001). Values are expressed as means ± SEM (n = 5–7).

### IMOs Preparation

The IMOs were prepared from maltodextrin by the transglycosylation activity of dextran dextrinase (EC 2.4.1.2) [23], which catalyzes the successive transfer of a glucosyl group from a terminal position in a dextrin molecule to a nonreducing terminal position in another molecule to make an α-1,6-glucosidic linkage. The products were thoroughly digested by hog pancreatic α-amylase (Sigma Chemical Company, USA) to reduce the maltodextrin portion incorporated into the synthesized saccharides. The carbohydrates in the mixture were fractionated with methanol precipitation. The precipitant with 40–90% methanol and the soluble fraction with 90% methanol were designated L-IMO and S-IMO, respectively. The components of IMOs were shown in Figure S1 and S2). Total sugar (TS) and reducing sugar (RS) of IMOs was determined by phenol sulfuric acid and 2,2′-bicinchoninate method, respectively. Their average degree of polymerization (DP) was calculated by TS/RS.

**Figure 2:**
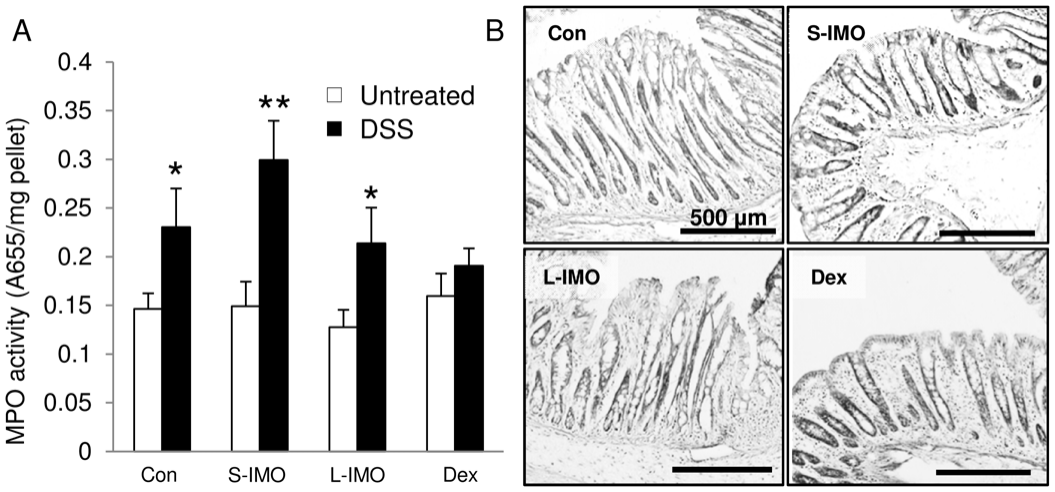
MPO activity and histological appearance. (A) MPO activity in the distal colon on day 7. Significant differences in activity between rats with or without DSS treatment were determined by an unpaired two-tailed Student’s *t*-test (*P<0.05, **P<0.01). Two-way ANOVA *P* values for MPO activity (diet and treatment) were <0.0001 for treatment. No significant difference was observed for treatment and the interaction between diet and treatment. Values are expressed as means ± SEM (n = 5–7). (B) Hematoxylin and eosin staining in a distal colonic section fixed with formalin. The scale bar represents 500 µm.

### DSS-induced Colitis

After acclimation for a week, the rats were fed the control, 3% S-IMO, 3% L-IMO, or 3% Dex diet for two weeks. In the last week, the rats were given 2% DSS (36–50 kDa; MP Biomedicals, Tokyo, Japan) in deionized water *ad libitum*. The rats were weighed daily and visually inspected for rectal bleeding and diarrhea. We determined the level of DSS-induced colitis using a disease activity index (DAI) [24]. Briefly, the scoring system included the examination of stool consistency, rectal bleeding, and weight loss (Table 2). On the last day of the experimental period, the rats were killed under pentobarbital anesthesia (Somnopentyl: Kyoritsu Seiyaku Co., Ltd., Tokyo, Japan; 50 mg/kg body weight). The colonic mucosa was scraped with a sterilized glass slide and stored at -80°C for the measurement of myeloperoxidase (MPO) activity.

**Figure 3:**
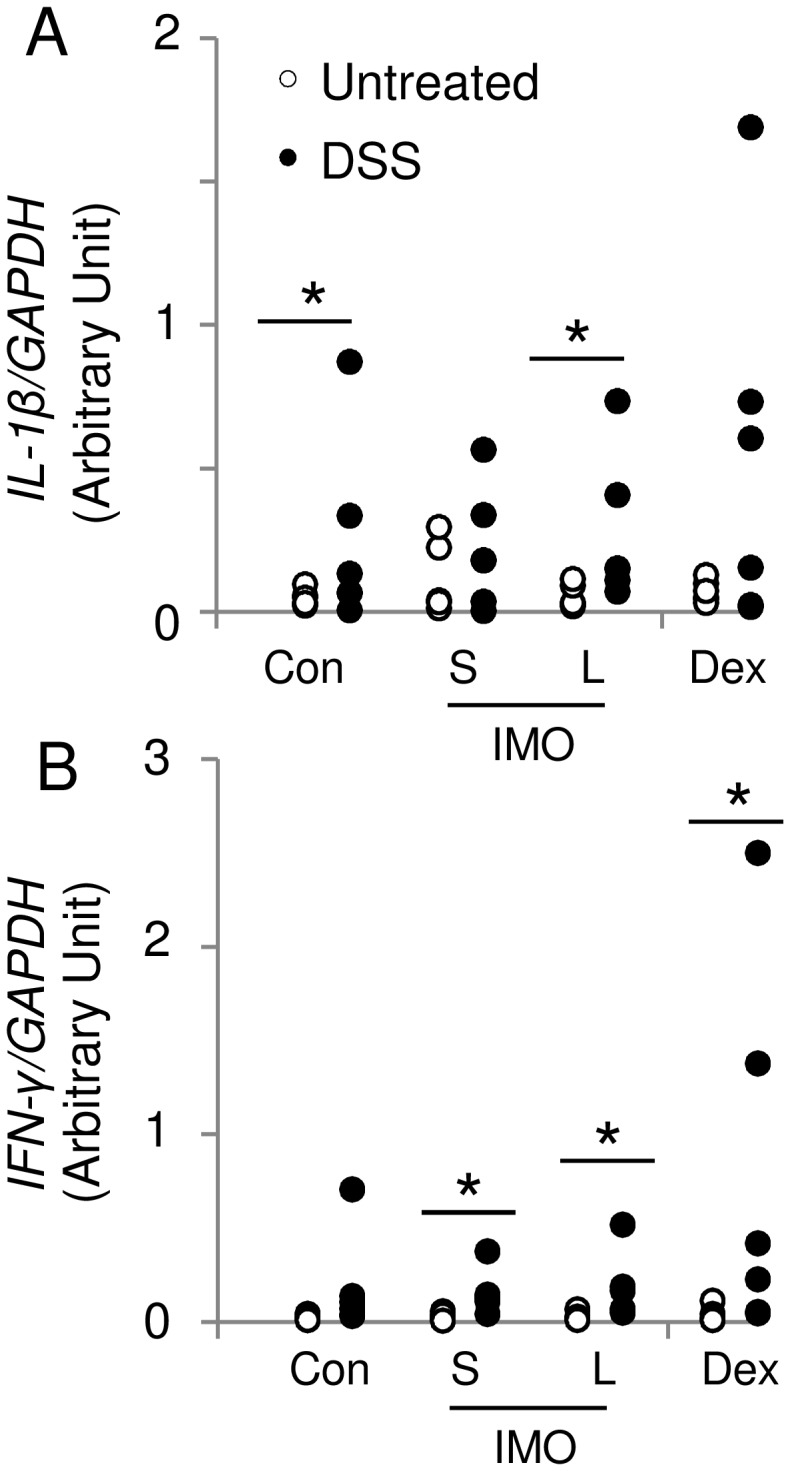
Inflammatory cytokine expression in distal colonic mucosa. The mRNA expression of *IL-1β* (A) and *IFN-γ* (B) was evaluated in the distal colon of rats with or without DSS treatment using a TaqMan gene expression assay. *GAPDH* was used as the internal control. Significant differences between rats with or without DSS treatment were determined by a median test (*P<0.05). Two-way ANOVA *P* values for *IL-1β* (A) and *IFN-γ* mRNA expression (diet and treatment) were 0.0042 and 0.0087 for treatment, respectively. No significant difference was observed for treatment and the interaction between diet and treatment. Values are expressed as means ± SEM (n = 6).

### MPO Activity

MPO activity was determined as described previously [25] and expressed as the increase in absorbance at 655 nm [ΔA_655_/(min·g pellet)].

**Table 3:** Cecal content weight, pH of cecal contents, and total SCFAs in cecal content of rats fed the control, S-IMO, L-IMO, or Dex diet.

	Con	S-IMO	L-IMO	Dex
**Cecal content weight (g)**				
Untreated	1.82±0.21	3.04±0.20^†^	1.99±0.34	4.02±0.40^†††^
DSS	2.35±0.45	4.23±0.88	2.54±0.46	5.86±0.35^**,†††^
**pH of cecal content**				
Untreated	7.81±0.03	7.16±0.04^†††^	7.76±0.02	7.17±0.18^†††^
DSS	7.24±0.12^***^	6.95±0.06^**^	7.38±0.08^***^	7.28±0.12
**Total SCFAs per cecal content (µmol/cecal content)**				
Untreated	79.5±6.9	171.2±25.0^††^	90.4±17.5	166.9±14.3^††^
DSS	46.0±14.2^*^	75.4±15.9^**^	63.8±17.0	121.8±10.9^*,††^

Values are expressed as means ± SEM (n = 6–7). Significant differences of the values the rats with or without 2% DSS treatment in each diet group (*P*<0.05) were determined by an unpaired two-tailed Student’s *t*-test (*P<0.05, **P<0.01, ***P<0.001). Significant differences of the values against control group in each treatment (*P*<0.05) were determined by Dunnett’s multiple comparison test (^†^ *P*<0.05, ^††^ *P*<0.01, ^†††^ *P*<0.01).

### Histochemistry

About 1 cm of excised distal colon was fixed with 10% formalin (v/v) in PBS at room temperature overnight, and the 10% formalin was then replaced with a 30% sucrose solution (w/v). The colonic tissues were embedded in optimal cutting temperature compound (Sakura Finetek Japan Co., Tokyo, Japan), frozen in liquid nitrogen, and stored at −80°C. The frozen colonic samples were sliced with a cryostat (Leica CM1500; Leica Microsystems, Wetzlar, Germany) into 7 µm thick sections. The sections were mounted on poly-L-lysine-coated (Sigma-Aldrich Co., St Louis, MO, USA) glass slides (Superfrost®; Matsunami glass Ind., Ltd., Osaka, Japan), stained with hematoxylin and eosin, and dehydrated. Histological images were captured using a microscope (IX81; Olympus, Tokyo, Japan) and DP Controller software (Olympus).

**Figure 4:**
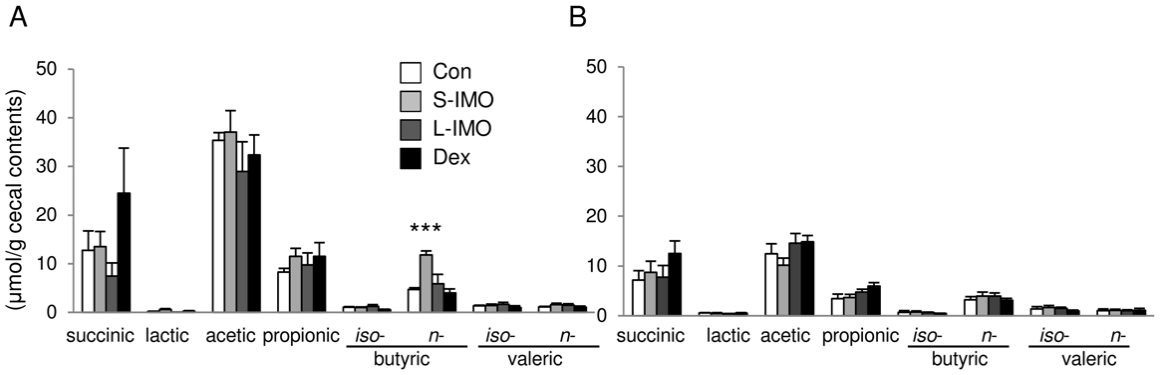
Concentrations of organic acids in cecal content. Organic acids (succinic, lactic, acetic, propionic, *iso*-butyric, *n*-butyric, *iso*-valeric and *n*-valeric acids) were measured via HPLC in the homogenates of cecal contents of (A) untreated or (B) DSS-treated rats. Significant differences in each organic acid were determined by Dunnett’s multiple comparison test (**P<0.01, ***P<0.001). Values are expressed as means ± SEM (n = 6).

### Analysis of mRNA Expression

The colonic mucosa (about 20 mg) was scraped with a sterilized glass slide and immediately frozen with liquid nitrogen. Frozen samples were kept -80°C until RNA extraction. Total RNA was extracted from the colonic mucosa using an RNeasy mini kit (Qiagen, Tokyo, Japan) according to the manufacturer’s instructions. The RNA concentration in a Tris-HCl buffer (pH 7.4) was measured by using spectrophotometry (SmartSpec™ Plus spectrophotometry; Bio-Rad, Hercules, CA, USA). We confirmed that the A260/A280 was higher than 1.8. The RNA (5 µg) was reacted with random hexamer primers (100 pmol; Toyobo Biologics, Inc., Osaka, Japan) at 70°C for 10 min in a final volume of 20 µL, followed by mixed with 2 µL of 1000 units of reverse transcriptase [Reverscript I M-MLV (RNaseH-); Wako], 8 µL of 5x Reaction buffer (Wako), 8 µL of 2.5 mmol/L dNTP mixture (Wako), and 2 µL of ribonuclease inhibitor (Ribonuclease inhibitor, human placenta, recombinant, solution; Wako) to convert to cDNA in a final volume of 40 µL at 37°C for 10 min and 98°C for 5 min. In this step, reverse transcriptase was replaced with nuclease free water for no-reverse transcription (RT) control. To check the genomic DNA contamination in samples, no-RT samples was amplified using GAPDH primers. We did not find any unexpected amplification in the no-RT samples in this experimental condition. The reaction mixture for quantitative PCR (qPCR) consisted of 2 µL of the cDNA template, 0.5 µL of primer and hydrolysis probe mixture(Taqman probe; Applied Biosystems, Foster City, CA, USA), 3.5 µL of nuclease free water (Promega, Madison, WI, USA), and 6 µL of Taqman universal PCR master mix (Applied Biosystems) in a final volume of 10 µL. Inhibition of PCR was checked by serial dilution of the sample. We used a diluted sample which did not interfere PCR reactions. The qPCR reaction mixtures were added to MicroAmp® Optical 96-Well Reaction Plate (Applied Biosystems). The samples into the wells of a microplate were sealed with Optically-clear adhesive film (MicroAmp® Optical Adhesive Film; Applied Biosystems). The qPCR reaction was performed using an Mx3000P real-time PCR system (Agilent Technologies, Santa Clara, CA) with TaqMan Gene Expression Assays for *IL-1β* (NM_031512, assay ID: Rn00580432_m1), *IFN-γ* (NM_138880, assay ID: Rn00594078_m1), and *GAPDH* (NM_017008, assay ID: Rn99999916_s1) as a reference gene. We confirmed that the mRNA expression of *GAPDH* correlated the total amount of mRNA in the sample and that any treatment in this experiment did not influence the correlation. The PCR consisted of a denaturing step at 95°C for 10 min, followed by 50 cycles of annealing with an extension step at 60°C for 30 sec and a denaturing step at 95°C for 20 sec. For each sample, the relative expression of mRNA was compared with that of *GAPDH* according to the standard-curve method. The baseline and quantification cycle were automatically determined using MxPro version 4.10 (Agilent Technologies). No template control was included for each primer pair, and each qPCR reaction was carried out in triplicate.

**Figure 5:**
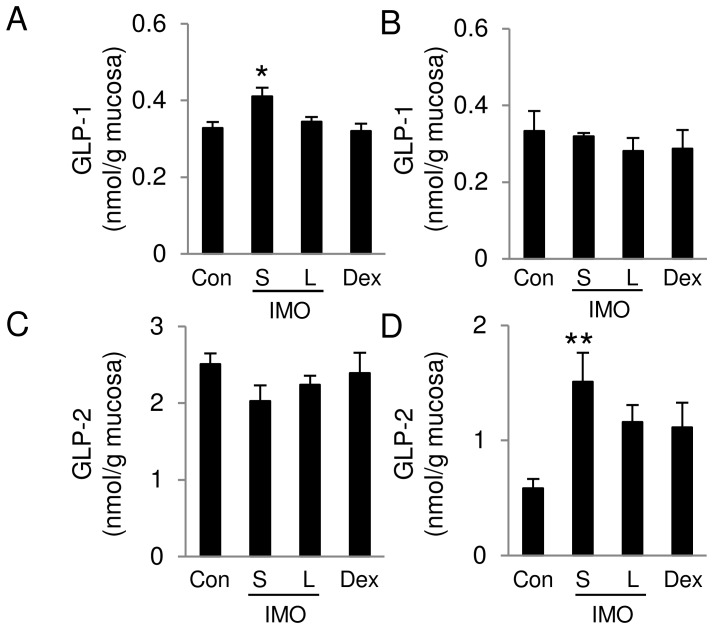
Concentrations of GLP-1 and GLP-2 in ileal and colonic mucosa. Active GLP-1 concentrations in the mucosa of (A) the terminal ileum and (B) the proximal colon were measured using a GLP-1 ELISA kit. Total GLP-2 concentrations in the mucosa of (C) the terminal ileum and (D) the proximal colon of rats treated with DSS were measured using a GLP-2 ELISA kit. Significant differences were determined by Dunnett’s multiple comparison test (*P<0.05, **P<0.01). Values are expressed as means ± SEM (n = 5–6).

### Measurement of Organic Acids

Cecal contents were diluted with four volumes of deionized water and homogenized. The pH of the homogenates was measured with a semiconducting electrode (ISFET pH sensor 0010–15C, Horiba Ltd., Kyoto, Japan). Organic acids (succinic, lactic, acetic, propionic, *iso*-butyric, *n*-butyric, *iso*-valeric and *n*-valeric acids) in the homogenates were analyzed by using HPLC (LC-10Adpv; Shimadzu Co., Ltd., Kyoto, Japan) equipped with two Shim-pack SCR-102H columns (8 mm i.d., 30 cm long; Shimadzu) and an electroconductibility detector (CDD-6A, Shimadzu) as described previously [26], [27].

**Figure 6:**
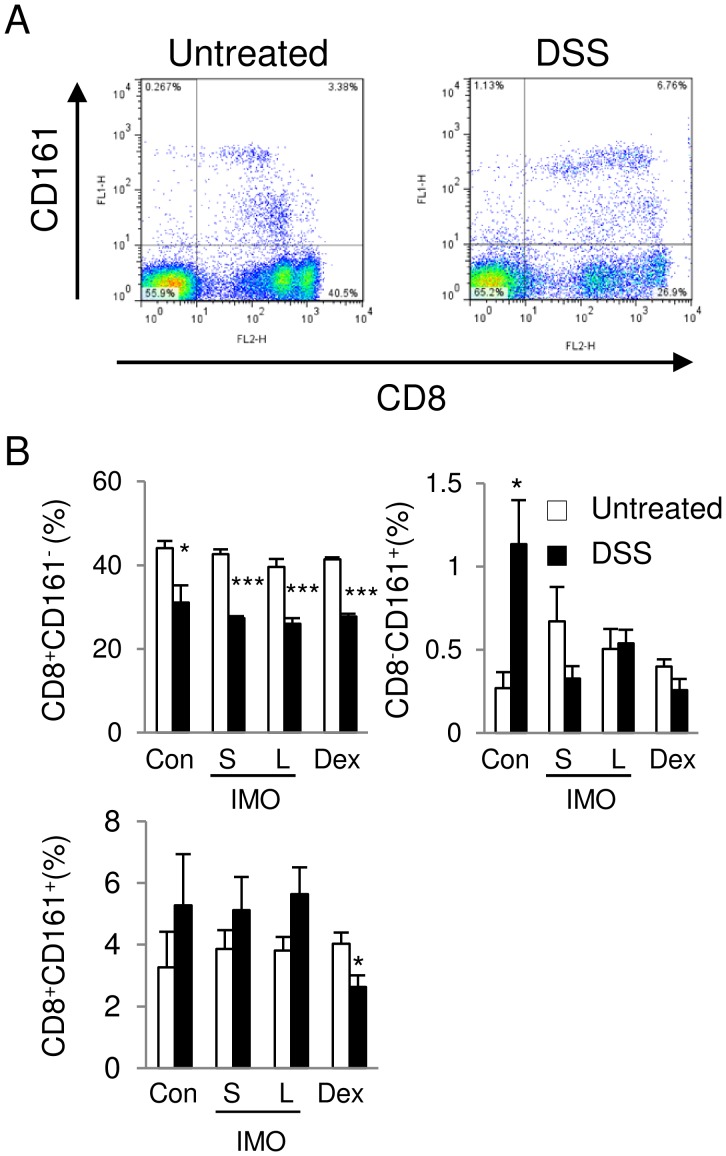
Phenotypic analysis of PBL. (**A**) Representative dot plots of phenotypes in PBL expressing CD8α and CD161 molecules in rats fed the control diet. (**B**) The percentage of CD8^+^ CD161^−^, CD8^−^CD161^+^, and CD8^+^CD161^+^ cells in the CD45^+^ cell populations. Significant differences between rats with or without DSS treatment were determined by an unpaired two-tailed Student’s *t*-test (*P<0.05, ***P<0.001). Values are expressed as means ± SEM (n = 3–5).

### Measurement of GLP-1 and GLP-2

Terminal ileum and proximal colon of rats treated with DSS were removed, and the contents were washed with PBS. About 100 mg of the tissue was collected, homogenized in an ethanol acid solution (74% ethanol, 0.12 mol/L HCl; 5 mL/g tissue), and incubated at 4°C for 24 h. The homogenate was centrifuged at 2,000×*g* for 30 min. The supernatant was collected and stored at −80°C until analysis. Active GLP-1 (7–36) and total GLP-2 were analyzed using ELISA kits (Glucagon Like Peptide-1 Active ELISA; Millipore Co., Billerica, MA, USA, and YK140 Rat GLP-2. EIA; Yanaihara Inc., Shizuoka, Japan).

### Population Analysis of Peripheral Blood Leukocytes (PBL)

Whole blood collected from the abdominal artery was treated with heparin sodium salt solution. The plasma was treated with ammonium chloride solution (pH 7.4; 8.26 g/L ammonium chloride, 1 g/L potassium bicarbonate, and 0.037 g/L EDTA-4Na) to lyse erythrocytes. PBS was added to neutralize the solution, and the solution was then centrifuged at 1,800×*g* for 20 min to separate the leukocyte fraction. The leukocyte fraction was suspended in PBS and passed through a cell strainer (35 µm nylon mesh; Becton, Dickinson and Company, Franklin Lakes, NJ, USA). The cell suspensions were stained with mouse anti-rat CD8α and CD161 monoclonal antibodies (AbD Serotec, MorphoSys UK, Oxford, UK) for 30 min at 4°C in darkness. The stained cells were washed twice, resuspended in PBS, and immediately analyzed using a FACSCalibur flow cytometer (Becton, Dickinson and Company). Data were collected and analyzed with FACS analysis software (FlowJo, version 7.7.2; TreeStar, Ashland, OR, USA).

### Statistics

Two-way ANOVA was used to evaluate in vivo differences in colon weight per length (diet and treatment), MPO activity, *IL-1β* mRNA expression, and *IFN-γ* mRNA expression. Dennett’s multiple comparison test was used to compare the values in each dietary group with those in the control group and to compare the daily DAI score values with those on day 0 in each dietary group. Differences between the untreated (without DSS) group and the 2% DSS-treated group were evaluated using an unpaired two-tailed Student’s *t*-test. Differences in cytokine expression between the untreated group and the 2% DSS-treated group were evaluated using a nonparametric median test. Statistical analysis was performed with JMP software (version 5.0; SAS Institute, Inc., Tokyo, Japan). Differences were considered statistically significant at *P*<0.05. All the values are presented as the mean ± SEM.

## Results

### S-IMO and Dex Diets Delayed Development of DSS-induced Colitis

Final body weight and food intake were not different among the four diet groups with or without DSS treatment (data not shown). A significant increase in DAI was found in rats fed the control and L-IMO diets on day 6 of the DSS exposure. In contrast, the ingestion of the S-IMO or Dex diets delayed the development of symptoms (Figure 1A). DSS administration significantly raised the ratio of the colonic weight per colon length, except for in the rats fed the Dex diet (Figure 1B).

### Ingestion of the Dex Diet Suppressed the Increase in MPO Activity

Seven days of DSS exposure significantly increased MPO activity in the colonic mucosa of rats fed the control, S-IMO, and L-IMO diets. There was no difference in MPO activity in rats fed the Dex diet (Figure 2A). Histological analysis revealed more severe damage in rats fed the control and L-IMO diets than in rats fed the S-IMO and Dex diets (Figure 2B).

### Modulation of Cytokine Expression by Various α-1,6-glucosaccharides

Seven days of DSS exposure significantly increased *IL-1β* mRNA expression in rats fed the control and L-IMO diets. In contrast, there was no difference in *IL-1β* mRNA expression in rats fed the S-IMO and Dex diets (Figure 3A). A significant increase in *IFN-γ* mRNA was observed in response to DSS administration in all groups except for the control group (Figure 3B).

### Ingestion of the S-IMO Diet Promoted N-butyric Acid Production in the Cecal Contents

Ingestion of the S-IMO and Dex diets significantly increased the weight of cecal contents and decreased the pH of the contents (Table 3). Commercially available isomaltooligosaccharides (average DP = 2.7, Isomalto 900P; Showa Sangyo Co., Ltd) did not increased cecal content weight (Figure S3). The concentration of *n*-butyric acid was higher in rats fed the S-IMO diet without DSS exposure than in rats fed the control diet (Figure 4A and B). Total SCFAs (sum of acetic, propionic, and *n*-butyric acid) were also increased in rats fed the S-IMO and Dex diets compared with the rats fed the control diet, especially in the untreated groups (Table 3). Organic acids concentration in cecal contents was comparable between the rats fed control diet and 3% Isomalto 900P-contained diet (Figure S3).

### Dietary S-IMO Promoted GLP-2 Production in the Proximal Colon

S-IMO increased active GLP-1 levels in the terminal ileum without affecting levels in the proximal colon (Figure 5A and B). In contrast, total GLP-2 levels were not different among the four groups in the terminal ileum. S-IMO significantly increased the total GLP-2 level in proximal colon (Figure 5C and D). Interestingly, we found that *GPR43* mRNA expression correlated with proglucagon only in rats fed the S-IMO diet (Figure S4), indicating that a relationship exists between GPR43 and proglucagon. DSS-induced colitis tended to decrease SCFA levels in cecal contents. The promotion of *GPR43* mRNA expression induced by dietary S-IMO is partially involved in the increase in GLP-2 production in colonic tissues, resulting in a delayed development of DSS-induced colitis in S-IMO-fed rats.

### Decrease in CD8^+^CD161^+^CD45^+^ Cell Populations in the PBL of Dex-fed Rats

Several reports show intestinal inflammation is consecutive to *in vivo* priming of CD8^+^ cytotoxic T lymphocytes [28]–[30] and CD161^+^ T cells releasing proinflammatory cytokines are frequently found in the intestinal mucosa [31]. Therefore, we thought that CD8^+^CD161^+^ population is associated with anti-inflammatory effects of S-IMO and Dex. DSS administration significantly decreased the percent of CD8^+^CD161^−^ cells in the CD45^+^ cell populations in all the groups. In contrast, the proportion of CD8^−^CD161^+^ cells increased in response to the DSS exposure in the control group. However, the S-IMO, L-IMO, and Dex diets suppressed the increase in the population. In rats fed the Dex diet, DSS exposure reduced the percentage of CD8^+^CD161^+^ cells (Figure 6A and B).

## Discussion

Dietary oligosaccharides influence intestinal fermentation and microbiota [2], [32], [33]. However, few attempts have been made to understand the physiological impacts of DP. In the present study, we demonstrated that the ingestion of an S-IMO diet (DP = 3.3) delayed the pathogenesis of DSS-induced colitis. Conversely, no improvement of colitis was observed when rats ingested L-IMO (average DP = 8.4). In addition, commercially available isomalto-oligosaccharides (DP = 2.7) containing isomaltose (33.4%), panose (8.5%), isomaltotriose (13.5%) did not show anti-inflammatory effect such as cecal fermentation (Figure S3), GLPs production in intestine (data not shown). The commercial isomalto-oligosaccharides component are mainly digested and absorbed in intestine without reaching large intestine, demonstrated by no increase in cecal content weight. These results indicate that even the small difference of DP has significant pathophysiological impacts.

Although the ingestion of the S-IMO and Dex diets delayed the development of colitis, the effects on the increases in ratio of colon weight per length and MPO activity were different between the rats fed the S-IMO diet and the rats fed the Dex diet in this experiment. Substantial PMN recruitment into the colonic tissue was further confirmed by the increased MPO activity in DSS-induced colitis [34]. The ingestion of the Dex diet suppressed the increases in the ratio of colon weight per length and MPO activity. In contrast, the ingestion of the S-IMO diet did not suppress these increases. We could not exclude the possibility that the ingestion of the Dex diet simply masked the DSS-induced colitis. In addition, the mechanism mediating the delayed development of DSS-induced colitis may be different between the S-IMO and Dex diets. This idea is supported by the differences in the CD8^+^CD161^+^ PBL populations. Lymphotoxins-related inducible ligand that competes for glycoprotein D binding to herpesvirus entry mediator on T cells (LIGHT), a cytokine in the TNF family, mediates inflammation specifically in the gut compartment when expressed on T cells [35], [36]. A recent study tested whether CD161 expression on peripheral blood T cells defines a gut-associated sub-population of cells that preferentially express LIGHT and demonstrated that CD8^+^CD161^+^ and CD8^+^CD161^−^ T cells express higher levels of LIGHT [37]. In addition, the neutralization of LIGHT reduces the severity of DSS-induced colitis similar to that in LIGHT-deficient mice [38]. Therefore, a reduction of CD161^+^CD8^+^ cells might contribute to the delayed development of colitis in rats fed the Dex diet. Dietary Dex possibly affected the population and functions of immune cells. We previously demonstrated that *IFN-γ* mRNA expression did not increase in early phase of DSS-induced colitis in an inbred strain originated from Wistar strain [39]. In contrast, *IL-1β* mRNA expression increased even in the early phase of the colitis. In our in-vitro experiment using peritoneal macrophages, we confirmed that IL-1β production was sharply stimulated by LPS (data not shown) within a couple of days. Other report also shows that the levels of IFN-γ in DSS-treated group were still within the range of those in untreated group on day 7 after start DSS administration, although IL-1β in the DSS-treated group has already increased on day 7 [40]. Further, anti-mouse IL-1β antibody suppressed clinical symptoms in murine acute DSS-induced colitis [41]. Therefore, we think that *IL-1β* mRNA expression is a key to understand the symptoms especially in an acute phase of colitis rather than *IFN-γ* mRNA expression. In the present study, we evaluated *IL-1β* and *IFN-γ* expression on only day 7 after start DSS administration. No significant increase in *IFN-γ* expression in rats fed control diet indicates that DSS might not increase yet on day 7 in line with other reports. The IMO and Dex are not digestible in small intestine and flow into large intestine. Macrophage or dendritic cell in intestine would incorporate these isomaltosaccharides and transfer them to lymph node. In our study, *IL-1β* mRNA expression tended to be slightly higher in the S-IMO and Dex diet group even without DSS treatment. Such increase in IL-1β might result in disappearance of expression difference in IL-1β in the S-IMO and Dex group.

In this study, the S-IMO diet particularly enhanced the cecal concentration of *n*-butyric acid, which has been reported to be a promising organic acid in the maintenance of intestinal homeostasis. Apart from being an important energy source for the colonocytes, butyrate directly modulates immune responses [42], [43]. Some reports show that butyric acid suppresses nuclear factor kappa B (NF-κB) activation [44], [45]. NF-κB is a transcription factor that controls the expression of genes encoding proinflammatory cytokines, chemokines, inducible inflammatory enzymes such as inducible nitric oxide synthase and cyclooxygenase-2, adhesion molecules, growth factors, some acute phase proteins and immune receptors [46]. A high concentration of butyric acid in cecal contents would contribute to an inhibition of the increase in *IL-1β* mRNA expression induced by DSS.

Butyrate and other SCFAs can act as signaling molecules through GPR41 and GPR43 [47]–[49]. *Gpr43* ^−/−^ mice showed greater morbidity in DSS-induced colitis [50]. A recent study revealed a direct association among GPR43 activation, elevation of intracellular Ca^2+^ in L-cells, and enhanced GLP-1 secretion from primary colonic cultures [18]. GLP-1 receptor activation reduces the accumulation of monocytes and macrophages and regulates inflammation in macrophages via the cAMP/PKA pathway and in monocytes via integrin-related gene expression [51]. GLP-2 stimulation improves mucosal healing in DSS- and indomethacin-induced intestinal inflammation [21], [52], [53]. Our results suggest that the enhancement of GLP-2 production in the proximal colon contributes to the amelioration of DSS-induced colitis in rats fed an S-IMO diet.

In summary, the S-IMO diet and Dex diet differentially delayed the development of DSS-induced colitis. The Dex diet may attenuate DSS-induced colitis by modulating immune cell population and functions. S-IMO contributes to the delayed development of colitis through fermentation in the large intestine. In addition, the GPR43 pathway is potentially associated with the amelioration of DSS-induced colitis by S-IMO. The mode of amelioration appears different between S-IMO and Dex diet. Therefore, the DP of α-1,6-glucosidic linkage is one determinant of the physiological impacts in experimental colitis. In addition, our data suggest that digestibility and linkage type in IMO component influence a functional benefit.

## Supporting Information

Figure S1 **1H NMR spectra of IMOs along with that of panose (A). Chemical structures of oligosaccharides (B).** Proton NMR spectra of IMOs were measured at 80°C in D_2_O using a JNM-AL400 spectrometer (JEOL Ltd., Tokyo, Japan). The sample concentration was 5 mg/mL. Sodium 4,4-dimethyl-4-sila-[2,2,3,3-D_4_]-pentanoate (TSP-*d*_4_) was used as the internal standard for the chemical shift. Those spectra were similar, and demonstrated a major difference in the signal intensity of peak **c1**. In the anomeric region, both S-IMO and L-IMO gave four peaks. Two signals observed at 5.27 and 4.89 ppm were assigned to anomeric protons (H-1) in a-(1→4) glycosidic linkages (**a**) and in a-(1→6) linkages (**c**), respectively, and the other two signals at 5.15 and 4.57 ppm were assigned to a-anomeric and b-anomeric protons in reducing ends (**b**), respectively. The peak area ratio of the four peaks at 5.27, 5.15, 4.89 and 4.57 ppm were 1.46∶0.47∶0.87∶0.53 for S-IMO and 2.38∶0.40∶5.16∶0.60 for L-IMO. S-IMO thus showed a molar ratio of reducing ends (**b**) to a-(1→6) linkages (**c**) was 1.00∶0.87, and it was 1.00∶5.16 for L-IMO. From the result, main components of IMOs were oligosaccharides having a structure **1** as shown below. The difference in the degree of polymerization between S-IMO and L-IMO was ascribed to the number (*n*) of a-(1→6) linkages (**c**) in the structure, that is, L-IMO has longer chains elongated with a-(1→6) linkages (**c**). HPAEC-PAD analysis revealed that both S-IMO and L-IMO contain panose, a trisaccharide having one a-(1→6) linkages (**c**) in the structure. We note that IMOs also contained a certain amount of other a-1,4 and a-1,6 glucooligosaccharides: compounds **2** (as shown below) as miner components. The presence of the oligosaccharides can be confirmed by a signal at 5.27 ppm (**d1**) belonging to H-1 of a-(1→4) linkages (**d**).
(TIF)

Figure S2 **MALD TOF spectra of S-IMO and L-IMO.** Degrees of polymerization of oligosaccharides are indicated at the peaks. The samples are dissolved in 9∶1 water-methanol containing 100 mM 2,5-dihydroxybenzoic acid. 2 mL of this mixture was applied to a MALD probe and dried. MALD-TOF spectra were recorded with a Bruker DALTONICS autoflex II spectrometer (Bruker Daltonics, Leipzig, Germany).
(TIF)

Figure S3 **The dietary effects of commercially available IMO.** MPO activity in colonic mucosa of rats fed control or isomalto 900P (DP = 2.7; Showa Sangyo Co., Ltd)-contained diet. (a). Cecal content weight of control and isomalto 900P groups with DSS. Organic acid concentration in cecal contents of rats fed control or isomalto 900P-contained diet without DSS (c) and with DSS treatment (d). Values are expressed as means ± SEM (n = 6). Significant differences in MPO activity between rats with or without DSS treatment were determined by an unpaired two-tailed Student’s *t*-test (*P<0.05). Significant differences in cecal content weight were determined by Dunnett’s multiple comparison test (vs. control; *P<0.05, **P<0.01). These values of control group are shared with Figure 2, 4 and Table 3.
(TIF)

Figure S4 **mRNA expressions of** ***GPR43*** **and** ***proglucagon*** **in distal colonic mucosa.** The mRNA expression of *GPR43* and *proglucagon* were evaluated in distal colonic mucosa of untreated rats. The cDNA was amplified using a specific primer pair for *glyceraldehydes-3-phosphate dehydrogenase (GAPDH)* (NM_0170008; forward: 5′-acattgttgccatcaacgac-3′, reverse: 5′-cacacccatcacaaacatgg-3′, annealing temperature 51°C, 360 bp), *GPR43* (NM_001005877; forward: 5′-tggaggctgtggtgtttc-3′, reverse: 5′-agctctgtgccccttctg-3′, annealing temperature 47.4°C, 207 bp) or *proglucagon* (NM_012707, forward: 5′-cattcacagggcacattcac-3′, reverse: 5′-ctctggtggcaaggttatcg - 3′, annealing temperature 55.0°C, 340 bp) in semi-quantitative RT-PCR analyses. mRNA expression of *GPR43* and *progulagon* againist *GAPDH* were analyzed by densitometry using ImageJ software (ver 1.45s; National institutes of Health, USA). GAPDH was used as the internal control. Significant differences between control (Con) diet group and the other diet group [short-sized isomaltooligosaccharides (S-IMO), long-sized IMO (L-IMO), dextran (Dex)] were determined by a multiple Wilcoxon test (*P*<0.05). The relationship between mRNA expression of *GPR43* and *proglucagon* was determined by calculating the Spearman correlation coefficient. Values are expressed as the means ± SEM (n = 5–6). The ingestion of Dex diet enhanced the *GPR43* mRNA expression. A similar tendency was observed in the rats fed S-IMO. The mRNA expression of *GPR43* strongly correlated with that of *proglucagon* in the rats fed S-IMO diet.
(TIF)
