# Supplementary figures and images for: The Delay in the Development of Experimental Colitis from Isomaltosyloligosaccharides in Rats Is Dependent on the Degree of Polymerization
Source: PLoS One. 2012 Nov 29;7(11):e50658. doi: 10.1371/journal.pone.0050658 (PMC3510184; doi:10.1371/journal.pone.0050658)

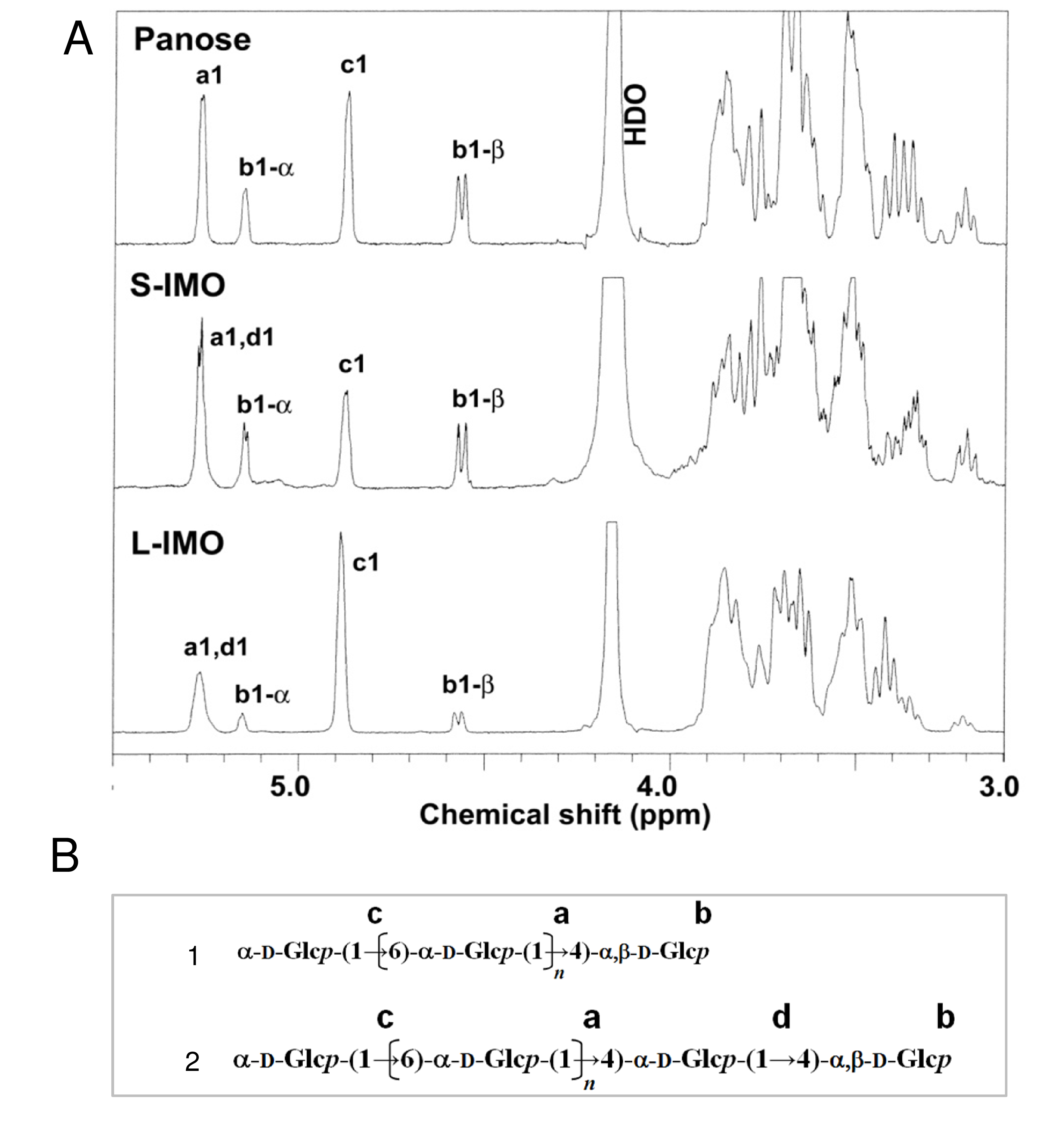

Supplement: Figure S1 — 1H NMR spectra of IMOs along with that of panose (A). Chemical structures of oligosaccharides (B). Proton NMR spectra of IMOs were measured at 80°C in D2O using a JNM-AL400 spectrometer (JEOL Ltd., Tokyo, Japan). The sample concentration was 5 mg/mL. Sodium 4,4-dimethyl-4-sila-[2,2,3,3-D4]-pentanoate (TSP-d 4) was used as the internal standard for the chemical shift. Those spectra were similar, and demonstrated a major difference in the signal intensity of peak c1. In the anomeric region, both S-IMO and L-IMO gave four peaks. Two signals observed at 5.27 and 4.89 ppm were assigned to anomeric protons (H-1) in a-(1→4) glycosidic linkages (a) and in a-(1→6) linkages (c), respectively, and the other two signals at 5.15 and 4.57 ppm were assigned to a-anomeric and b-anomeric protons in reducing ends (b), respectively. The peak area ratio of the four peaks at 5.27, 5.15, 4.89 and 4.57 ppm were 1.46∶0.47∶0.87∶0.53 for S-IMO and 2.38∶0.40∶5.16∶0.60 for L-IMO. S-IMO thus showed a molar ratio of reducing ends (b) to a-(1→6) linkages (c) was 1.00∶0.87, and it was 1.00∶5.16 for L-IMO. From the result, main components of IMOs were oligosaccharides having a structure 1 as shown below. The difference in the degree of polymerization between S-IMO and L-IMO was ascribed to the number (n) of a-(1→6) linkages (c) in the structure, that is, L-IMO has longer chains elongated with a-(1→6) linkages (c). HPAEC-PAD analysis revealed that both S-IMO and L-IMO contain panose, a trisaccharide having one a-(1→6) linkages (c) in the structure. We note that IMOs also contained a certain amount of other a-1,4 and a-1,6 glucooligosaccharides: compounds 2 (as shown below) as miner components. The presence of the oligosaccharides can be confirmed by a signal at 5.27 ppm (d1) belonging to H-1 of a-(1→4) linkages (d). (TIF) [file pone.0050658.s001.tif]

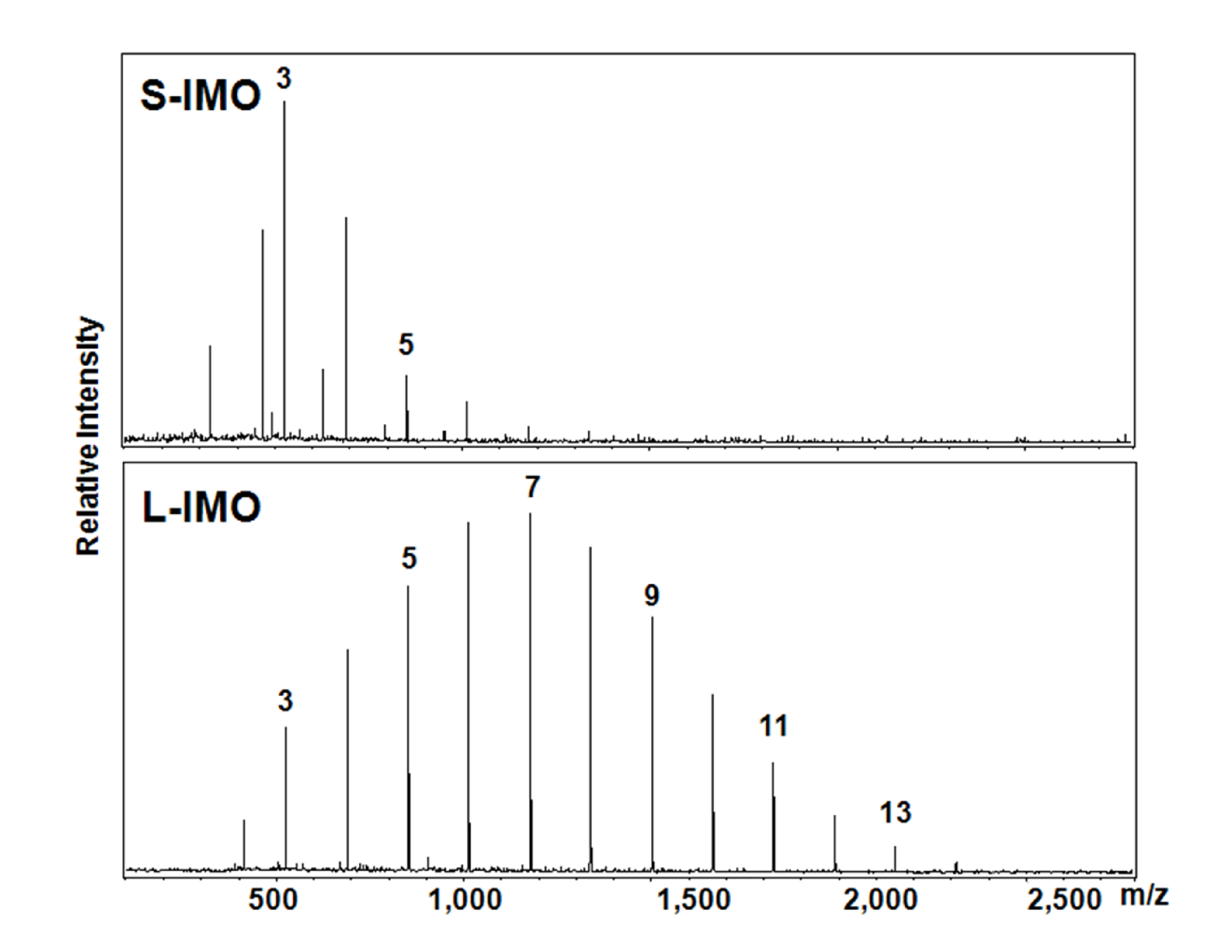

Supplement: Figure S2 — MALD TOF spectra of S-IMO and L-IMO. Degrees of polymerization of oligosaccharides are indicated at the peaks. The samples are dissolved in 9∶1 water-methanol containing 100 mM 2,5-dihydroxybenzoic acid. 2 mL of this mixture was applied to a MALD probe and dried. MALD-TOF spectra were recorded with a Bruker DALTONICS autoflex II spectrometer (Bruker Daltonics, Leipzig, Germany). (TIF) [file pone.0050658.s002.tif]
